# Tunable Duplex Metalens Based on Phase-Change Materials in Communication Range

**DOI:** 10.3390/nano9070993

**Published:** 2019-07-10

**Authors:** Wei Bai, Ping Yang, Shuai Wang, Jie Huang, Dingbo Chen, Zhaojian Zhang, Junbo Yang, Bing Xu

**Affiliations:** 1 The Key Laboratory of Adaptive Optics, Chinese Academy of Sciences, Chengdu 610209, China; 2 Center of Material Science, National University of Defense Technology, Changsha 410073, China; 3 Institute of Optics and Electronics, Chinese Academy of Sciences, Chengdu 610209, China; 4 University of Chinese Academy of Sciences, Beijing 100049, China

**Keywords:** duplex metalens, tunable, phase change materials, phase modulation, nano device

## Abstract

Metalenses recently have attracted attention because of their more compact size in comparison with conventional lenses; they can also achieve better optical performance with higher resolution. Duplexer is an interesting function of a metalens that can distinguish different sources and divide them into two parts for specific purposes. In this article, we design tunable duplex metalenses with phase-change material Ge_2_Sb_2_Te_5_ for the first time. Two types of special unit cells are designed to modulate the incident lights, and four metalenses are designed based on the two types of unit cells. Specific phase profiles are calculated for different sections of metalens in which the corresponding unit cells are settled; accordingly, the metalenses can focus the incident lights at any positions according to our design. Moreover, the metalenses become selectable via tuning the state of phase-change material, which means that the output light field can be actively controlled. The proposal of our tunable duplex metalenses will offer new opportunities for active three-dimensional imaging or optical coding.

## 1. Introduction

Recently, researchers have begun to pay increasing attention to metasurfaces due to their extraordinary performance in electromagnetism fields [1,2,3,4,5,6,7,8,9,10,11,12,13,14,15,16,17,18,19,20,21]. As 2D, artificial, photonic metamaterials, metasurfaces can manipulate the electromagnetic wave freely. By shaping the subwavelength resonators of metasurfaces, the amplitude, phase, polarization, and propagation direction of light can be easily controlled [1,2,3,4]. With the completion of new theories and technologies in this field, they have been applied to various functions, such as to ultrathin metalens [5,6], cloaking [7,8], nonlinear device [9,10], holograms [11,12], surface plasmon launcher [13], computing [14,15], biosensing [16,17], switching [18,19] and many novel photonic devices [20,21]. Metalens has become an exciting research topic that holds great promise to the applications of metasurfaces. Metalens can not only show better optical performance but also be smaller and lighter, which is more suitable for compact devices in comparison with conventional lens. Recently, excellent research about metalens have been reported. For example, Khorasaninejad et al. verified the exceptional ability of metalens experimentally [5]. Also, other functions such as chromatic aberration correction [6,22,23,24] and active tuning [25,26,27] have been proposed or proved. However, there are still various functions to be achieved, owing to the subwavelength manipulation to the electromagnetic field.

Duplexer can divide a light source into two parts for distinct uses. Using this as inspiration, we can design duplex metalens that can separate light sources and focus or image them at designed positions, which is impossible for conventional lenses. This is especially important in beam splitter or three-dimensional imaging. Some previous efforts have explored the novel functions of metalens. For instance, Li et al. focused light with different wavelengths into different positions [28], and Boroviks et al. focused different polarized lights into different positions [29]. Nevertheless, once metalenses are fabricated, the functions are fixed, which makes them inflexible. However, this situation can be improved with the help of tunable phase-change materials.

Phase-change materials have been applied in optical disk storage for many years. Recently, they have been increasingly used for electrical nonvolatile memories in which their refractive index can be selectively tuned [30,31,32]. As a typical phase-change material, Ge_2_Sb_2_Te_5_ (GST) alloy has two different states: the amorphous state and the crystalline state. Different phase states correspond to different lattice arrangements such as amorphous, metastable, face-centered cubic (Fcc), and stable hexagon, all of which result in great complex permittivity contrast at near-infrared (NIR) and middle-infrared (MIR). Especially, the state transition between amorphous and crystalline can be precisely controlled by appropriate thermal, optical, or electrical stimuli [30,31,32]. The amorphous GST is gradually crystallized when it is heated to the temperature between the transition temperature (160 °C) and the melting temperature (627 °C) [33]. After a short, high-density laser pulse melts the GST and is it quickly quenched, the crystallized GST returns to an amorphous state (the reamorphization temperature is 640 °C) [34]. GST has become the most promising candidate for the next generation of controllable, nonvolatile photonic devices due to its ultrafast switching speed (occurring in a nanosecond or less) [32], high-switching stability (potentially up to 10^15^ cycles) [35], and compatibility with Complementary Metal Oxide Semiconductor (CMOS) technology. Lately, the combination of GST with metasurfaces has attracted increasing interest because active control is desirable in many nano-photonic devices. Several metadevices, such as display [36], filter [37], absorber [38], beam steering [39], modulation [40,41,42] and other novel devices [34,43,44,45,46], all of which are based on phase change materials, have been investigated. All devices have demonstrated the great potentialities of GST in active and flexible control of nano-devices.

In this work, we design the tunable duplex metalens based on phase-change material GST in the communication wavelengths (1.55 μm and 1.31 μm) for the first time. Two unit cells and four metalenses have been designed to test our method. The metalenses are composed of a series of well-designed GST nanocube resonators accompanying Pancharatnam-Berry (P-B) phase shift. Importantly, the metalenses were divided into two sections, and different sections were arranged with different unit cells to achieve duplex function. When the GST structures remained at the amorphous state, the corresponding light was able to pass through. When the GST structures stayed at the crystalline state, the corresponding light was shut off. Accordingly, we can selectively control the focused light on demand by adjusting the state of GST when the incident light is 1.31 μm, 1.55 μm, or both. We believe that the proposed tunable duplex metalens could be applied in active three-dimensional imaging or optical coding.

## 2. Single-Wavelength Tunable Duplex Metalenses

**Figure 1:**
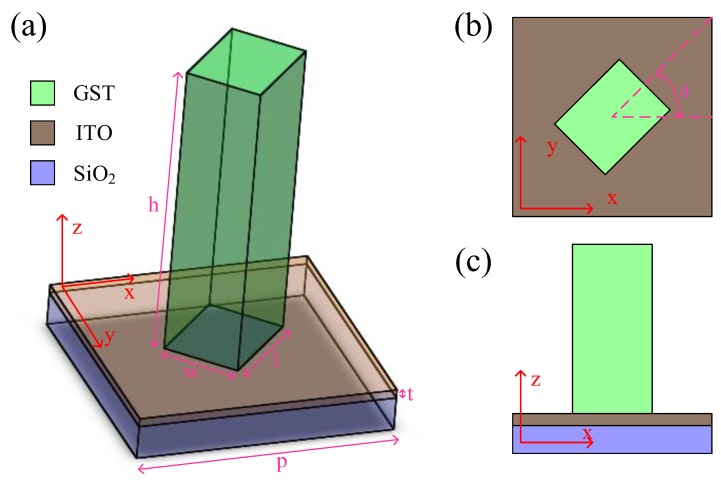
(**a**) Overview of the unit cell. The structured GST with period *p*, height *h*, length *l*, width *w* is settled on the substrate SiO_2_. A 30-nm thin film ITO is deposited between the GST and substrate as a conductive layer. (**b**) Top view of the unit cell. The structured GST forms an angle *θ* with *x* axis. (**c**) Side view of the unit cell.

The structure of the unit cell is depicted in Figure 1a. A nanocuboid waveguide GST was placed on the transparent SiO_2_ substrate. The orientation of GST formed an angle *θ* with *x* axis as shown in Figure 1b; Figure 1c is the side view of the unit cell. A 30-nm thin transparent film of indium tin oxide (ITO) was sandwiched between the GST and the SiO_2_ substrate. This film was treated as a conductive layer to tune the state of GST by electrically heating the structured GST. 

In this work, P-B phase shift is adopted to achieve phase control. A left/right circularly polarization (LCP/RCP) incident light passes through the unit cell, propagating along *+z* direction; by applying the Jones matrix theory, the output field can be simplified as [28]:

(1) $$\begin{array}{cc} E_{out} & =\alpha \left[\begin{array}{cc} cos^{2}\theta & sin\theta cos\theta \\ sin\theta cos\theta & sin^{2}\theta \end{array}\right]E_{L/R}=\alpha \left[\begin{array}{cc} cos^{2}\theta & sin\theta cos\theta \\ sin\theta cos\theta & sin^{2}\theta \end{array}\right]\frac{1}{\sqrt{2}}\left[\begin{array}{c} 1\\ \pm i \end{array}\right]\\ & =\frac{1}{2\sqrt{2}}\alpha \left(cos2\theta +i*sin2\theta \right)\left[\begin{array}{c} 1\\ \mp i \end{array}\right]+\frac{1}{2\sqrt{2}}\alpha \left[\begin{array}{c} 1\\ \pm i \end{array}\right]\\ & =\frac{1}{2\sqrt{2}}\alpha e^{i*2\theta }E_{R/L}+\;\frac{1}{2\sqrt{2}}\alpha E_{L/R} \end{array}$$

Here, the incident light $E_{L/R}=1/\sqrt{2}\left(e_{x}\;\pm \;i*e_{y}\right)$ is the normalized representation of Jones matrix for LCP and RCP separately, and $\alpha =\;t_{o}\pm t_{e}$, where *t_o_* and *t_e_* represent the complex transmission coefficients along the optical fast axis and slow axis which is determined by the parameters of the unit cell (*p*, *l*, *w*, *h*, *ε*(*ω*)), and the symbol “±” distinguishes the co-polarization and cross-polarization light, respectively. According to the formula, the incident CP light is divided into two cross polarization outputs. The co-polarization light only receives the amplitude modulation but the cross-polarization light has the 2θ phase modulation simultaneously. 2π phase tuning can be achieved for the cross-polarization light if the angle θ rotates from 0 to π. When the state of the GST is changed, the transmission is changed simultaneously; thus the output of the unit cell can be actively controlled.

**Figure 2:**
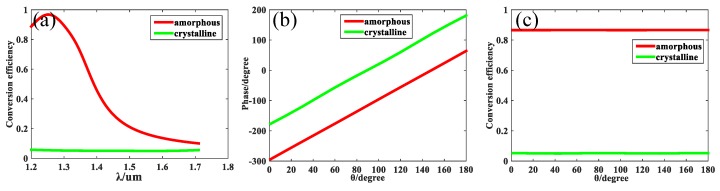
(**a**) The conversion efficiency from LCP to RCP versus wavelength for unit cell U_1_ when GST stays in amorphous state (red line) and crystalline state (greed line). (**b**) The phase modulation of unit cell U_1_ versus rotating angle *θ* for both states of GST at the designed wavelength of 1.31 μm. (**c**) The conversion efficiency of unit cell U_1_ from LCP to RCP versus rotating angle *θ* for both states at the designed wavelength of 1.31 μm.

The simulation of all the unit cells was performed using finite-element method in frequency domain with the unit cell boundary in *xy* direction and the open boundary in *z* direction; the minimum size of the mesh was 20 nm. All unit cells were settled in free space. The information of the system used for computing this structures is as follows: The CPU of the system was Intel(R) Xeon(R) CPU E5-2420 v2; the RAM was 24 GB; the duration of the calculation for one unit cell was approximately several minutes; the HDD space was 1.72 TB. In the simulation, the incident LCP plane wave propagated along +*z* direction through the substrate. The GST data were obtained from the experimental data in reference [47], in which the dielectric function *ε(ω*) was investigated by infrared spectroscopy and spectroscopic ellipsometry. The conversion efficiency from LCP to RCP and the phase modulation of the unit cell were calculated from the simulations on the unit cell. After optimization at the wavelength of 1.31 μm, the parameters of the unit cell U_1_ were determined to be *p* = 600 nm, *h* =700 nm, *l* = 230 nm and *w* = 180 nm. According to the theoretical analysis from Equation (1), a part of incident light will be changed into RCP light with phase modulation of φ=2θ. When θ=0, the conversion efficiency from LCP to RCP for two different GST states is shown in Figure 2a. When the GST remained at amorphous state, the conversion efficiency was as high as 87% at the wavelength of 1.31 μm; in contrast, the conversion efficiency was only 16% at the wavelength of 1.55 μm (used for another purpose). When the GST was in crystalline state, the *ε(ω)* changed and the conversion efficiency was tuned lower than 5%, which can be treated as turning off in the whole range from 1.31 μm to 1.55 μm. Next, the incident light was fixed at 1.31 μm, at which point the angle of structure was rotated from 0 to π; the corresponding phase modulation is shown in Figure 2b. For amorphous GST, the simulation showed no difference with theoretical analysis; however, the phase modulation linearly covered the entire 2π range. Figure 2c shows that the conversion efficiency remained largely unchanged when the angle was rotated from 0 to π, which indicates that the angle had little influence in the conversion efficiency for two different states of GST. All simulations demonstrated that the unit cell is a good candidate for tunable metalens.

To focus like a conventional spherical lens, the phase profile of the metalens should satisfy:

(2) $$\varphi (x,y,f)=-\frac{2\pi }{\lambda }(\sqrt{{\left(x-x_{d}\right)}^{2}+{\left(y-y_{d}\right)}^{2}+f^{2}}-f)$$

Where *λ* is the designed wavelength, *f* is the focal length. (*x*, *y*, *0*) represents the position of the unit cell, and (*x_d_*, *y_d_*, *f*) represents the coordinate of an arbitrary focal point. According to the simulation result of *φ* = 2*θ*, the rotating angle of the unit cell in (*x*, *y*, *0*) should be:

(3) $$\theta (x,y,f)=-\frac{\pi }{\lambda }(\sqrt{{\left(x-x_{d}\right)}^{2}+{\left(y-y_{d}\right)}^{2}+f^{2}}-f)$$

**Figure 3:**
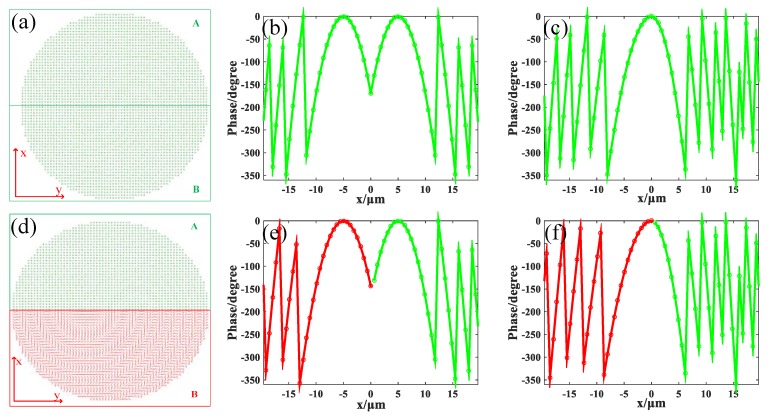
(**a**) The layout of the single wavelength tunable duplex metalens (M_1_, M_2_). The metalens is divided into two equal sections A and B, composed of unit cells U_1_. (**b**) The phase profile of metalens M_1_ along the *x* axis while *y* = 0, the central position of the metalens is at (0, 0). (**c**), (**e**) and, (**f**) adhere to the same conditions. (**c**) The phase profile of metalens M_2_. (**d**) The layout of the dual wavelength tunable duplex metalens (M_3_, M_4_). Section A is constructed with unit cells U_1_ and section B is constructed with unit cells U_2_. (**e**) The phase profile of metalens M_3_. (**f**) The phase profile of metalens M_4_.

The designed metalens is divided into two equal sections marked as A (*x* > 0) and B (*x* < 0). Unit cells U_1_ were used to construct the metalens M_1_. Specially, unit cells in section A were arranged as *x_d_* = 5 μm, *y_d_* = 0, *f* = 20 μm; unit cells in section B were arranged as *x_d_* = −5 μm, *y_d_* = 0, *f* = 20 μm. The radius of the metalens was fixed at 20 μm. The overall structure layout is depicted in Figure 3a. Figure 3b shows the imperfect phase distribution of metalens M_1_ along the *x* axis sampling at the center of each unit cell.

The results of all the metalenses were simulated by using finite integrity in time domain and open boundary condition in all directions; the size of the uniform mesh was set at 20 nm along all axes to minimize numerical errors. The entire devices were settled in free space. The duration of the calculation for one metalens was about 24 h (different metalenses spend different time). The light source was set at 1.31 μm LCP plane wave in the simulation. The spatial dimension of incident light is 40 × 40 μm^2^ and the LCP plane wave can be expressed as:

(4) $$E=\cos\left(kz-\omega t\right)\vec{e_{x}}+cos\left(kz-\omega t+\pi /2\right)\vec{e_{y}}$$

The incident light was settled at the position lower 1 μm from the metalens. The thickness of the substrate, ITO layer, and GST was 0.2 μm, 0.03 μm, and 0.7 μm, respectively. The output light field was obtained from the position upper 1 μm from the metalens. The spatial dimension of the simulation domain for the full metalens is 40 × 40 × 2.93 μm^3^. The light field was then exported after finite integrity in time domain and calculated by Matlab with Angular Spectrum Diffraction method in the spatial dimension of 40 × 40 × 40 μm^3^.

**Figure 4:**
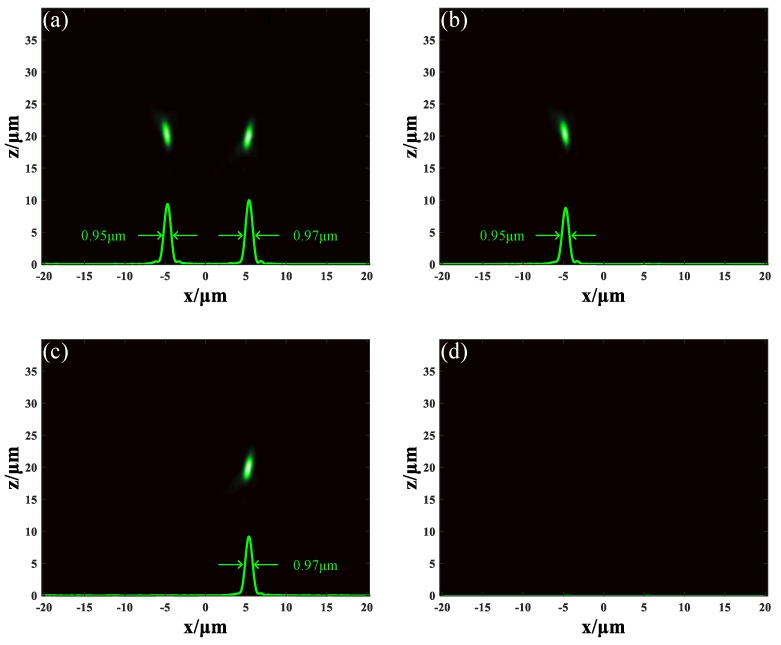
The light field distribution of metalens M_1_. (**a**) The GST in both sections are in amorphous state. (**b**) The GST in section A stays in amorphous state but the GST in section B stays in crystalline state. (**c**) The GST in section A stays in crystalline state but the GST in section B stays in amorphous state. (**d**) The GST in both sections are in crystalline state.

When the GST in both sections remained in an amorphous state, the incident light was separated into two parts: one focused at the position (5.29, 0.32, 19.9) μm, the other focused at the position (−4.64, 0.32, 20.3) μm. The result is illustrated in Figure 4a. Full width at half maximum (FWHM) of the spots in focal plane were 0.95 μm and 0.97 μm, respectively, which indicates that the metalens achieved subwavelength resolution. The deviance from the expected result (±5, 0, 20) μm came from the discrete phase distribution, as depicted in Figure 3b. By applying appropriate electrical current pulse through the ITO layer, the state of the GST can be changed. When the GST in section A remained in an amorphous state and the GST in section B was changed into a crystalline state, the incident light was focused at the position (−4.64, 0.32, 20.3) μm with FWHM of 0.95 μm, as shown in Figure 4b. When the GST in section A was changed into a crystalline state while the GST in section B was in an amorphous state, the incident light was focused at the position (5.29, 0.32, 19.9) μm with FWHM of 0.97 μm, as shown in Figure 4c. The result of the GST in both sections changing to a crystalline state is shown in Figure 4d. There was no light focusing because the conversion efficiency from LCP to RCP is very low when GST remains in a crystalline state. The results prove that the focusing effect of our tunable duplex metalens can be actively controlled in horizontal direction and will bring some convenience to integrated optical systems.

**Figure 5:**
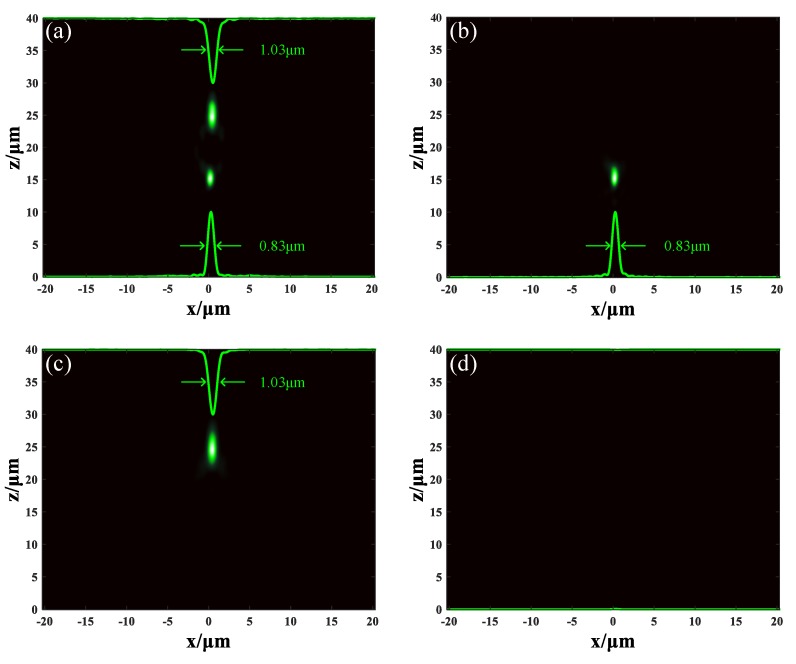
The light field distribution of metalens M_2_. (**a**) The GST in both sections are in amorphous state. (**b**) The GST in section A stays in amorphous state, but the GST in section B stays in crystalline state. (**c**) The GST in section A stays in crystalline state, but the GST in section B stays in amorphous state. (**d**) The GST in both sections are in crystalline state.

Although metalens M_1_ was designed to have the focus spots arranged in horizontal, in this study, we designed metalens M_2_ to have the focus spots arranged in vertical. Metalens M_2_ was also constructed by unit cells U_1_. Differently, unit cells in section A were arranged as *x_d_* = 0, *y_d_* = 0, *f* = 15 μm, and unit cells in section B were arranged as *x_d_* = 0, *y_d_* = 0, *f* = 25 μm. The phase distribution of metalens M_2_ along the *x* axis is shown in Figure 3c. The conditions of simulation remained unchanged, and the incident light remained 1.31 μm LCP plane wave. When the GST in both sections remained in an amorphous state, the incident light was focused at two different positions: (0.27, 0.27, 15.2) μm and (0.35, 0.35, 24.7) μm. The distribution of light field is depicted in Figure 5a. FWHM of the spot at z = 15.2 μm was 0.83 μm and FWHM of the spot at z = 24.7 μm was 1.03 μm. This is reasonable because longer focal length means smaller numerical aperture (NA), resulting in a larger focal spot. When the GST in section A stayed in an amorphous state and the GST in section B changed to a crystalline state, the incident light was focused at the position (0.27, 0.27, 15.2) μm with FWHM of 0.83 μm, as shown in Figure 5b. When the GST in section A was changed into a crystalline state but the GST in section B remained in an amorphous state, the incident light was focused at the position (0.35, 0.35, 24.7) μm with FWHM of 1.03 μm, as shown in Figure 5c. When the GST in both sections was changed into crystalline state, the metalens was turned off completely, as seen in Figure 5d. These results demonstrate that our tunable duplex metalens can actively control the focusing in vertical direction, and it may have potential in three-dimension imaging.

## 3. Dual-Wavelength Tunable Duplex Metalenses

**Figure 6:**
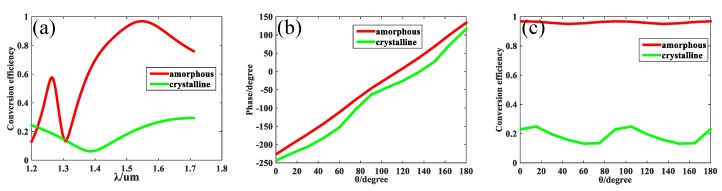
(**a**) The conversion efficiency from LCP to RCP versus wavelength for unit cell U_2_ when GST stays in different states. (**b**) The phase modulation of unit cell U_1_ versus rotating angle *θ* for different states of GST at the designed wavelength of 1.55 μm. (**c**) The conversion efficiency of unit cell U_1_ from LCP to RCP versus rotating angle *θ* for different states of GST at the designed wavelength of 1.55 μm.

When incident light becomes a mixed light of 1.55 μm and 1.31 μm, one single structure apparently can not accomplish the task to distinguish the two different frequencies of lights. Accordingly, another unit cell, U_2_, needs to be designed to modulate the phase at wavelength of 1.55 μm. The parameters of U_2_ are determined to be *p* = 600 nm, *h* = 700 nm, *l* = 505 nm, and *w* = 100 nm after optimization. The same period and height as unit cell U_1_ was selected to make sure that the different unit cells can be fabricated in the same steps. In this simulation, the LCP plane wave passed through U_2_ along *+z* direction. When the angle *θ* = 0, the conversion efficiency from LCP to RCP for two different states of GST is shown in Figure 6a. When GST stayed at amorphous state, the conversion efficiency was as high as 97% at the wavelength of 1.55 μm; however, the conversion efficiency was only 23% at the wavelength of 1.31 μm. The two unit cells U_1_ and U_2_ were designed specially; U_1_ let the 1.31 μm light pass but turned off the 1.55 μm light whereas U_2_ let the 1.55 μm light pass but turned off the 1.31 μm light, which will be very useful to separate multi-wavelength incident lights. When the GST stayed in crystalline state, the conversion efficiency was lower than 23% in the range from 1.31 μm to 1.55 μm, which can be treated as turning off in comparison with the effect of the amorphous GST. Next, the incident light was fixed at the wavelength of 1.55 μm. The angle of the structure was rotated from 0 to π, affecting the corresponding phase modulation, as depicted in Figure 6b. The results fit well with theoretical analysis because the phase modulation covers the whole 2π range linearly when the GST stays in amorphous state. Figure 6c demonstrates the relationship between conversion efficiency and the rotating angle, and the results prove that the rotating angle has no influence on the conversion efficiency for unit cell U_2_. 

Metalens M_3_ is constructed by unit cells U_1_ and U_2_ together. Unit cells U_1_ are arranged in section A as *x_d_* = 5 μm, *y_d_* = 0, *f* = 20 μm, and unit cells U_2_ are arranged in section B as *x_d_* = −5 μm, *y_d_* = 0, *f* = 20 μm. Figure 3d is the overview of the metalens, in which the green structures represent U_1_ and the red structures represent U_2_. The phase distribution of the metalens M_3_ along the *x* axis is depicted in Figure 3e.

**Figure 7:**
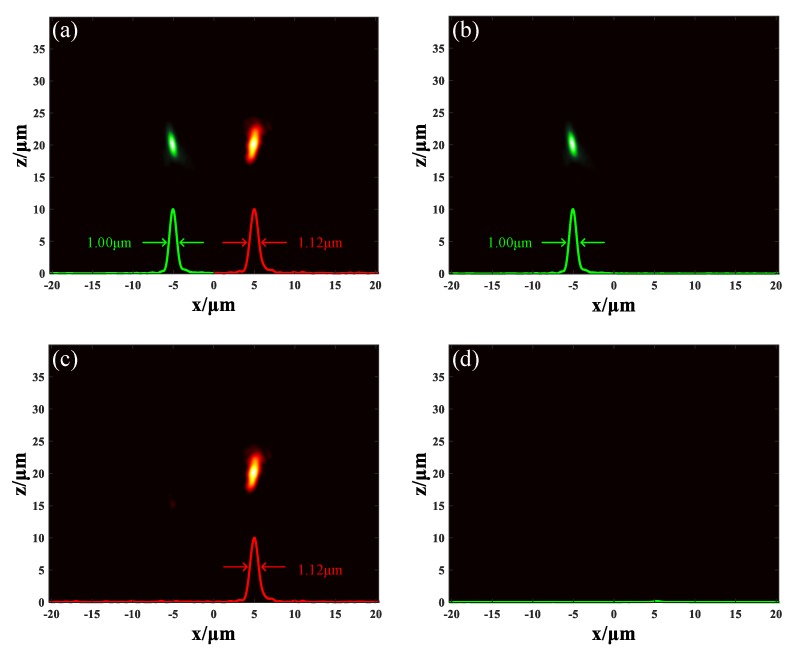
The light field distribution of metalens M_3_. The red profile and green profile indicate the 1.55 μm and 1.31 μm light respectively. (**a**) The GST in both sections are in amorphous state. (**b**) The GST in section A stays in amorphous state but the GST in section B stays in crystalline state. (**c**) The GST in section A stays in crystalline state but the GST in section B stays in amorphous state. (**d**) The GST in both sections are in crystalline state.

In this simulation, the conditions remain unchanged except the incident light source was changed into mixed light of two wavelengths at 1.55 μm and 1.31 μm. When the GST in both sections stayed in an amorphous state, the unit cells in section A and B could allow the light of 1.55 μm and 1.31 μm to pass through, respectively. The incident 1.55 μm light was focused at the position (4.93, 0.38, 20.2) μm with FWHM of 1.12 μm and the incident 1.31 μm light was focused at the position (−4.96, 0.38, 20.1) μm with FWHM of 1.00 μm, which have been marked as red spot and green spot, respectively, as seen in Figure 7a. The size of the spot was proportional to the wavelength while the focal lengths were equal. When the GST in section A stayed in an amorphous state but the GST in section B changed into a crystalline state, only the 1.31 μm incident light was focused at the position (−4.96, 0.38, 20.1) μm with FWHM of 1.00 μm, as shown in Figure 7b. When the GST in section A changed to a crystalline state but the GST in section B was in an amorphous state, the 1.55 μm incident light was focused at the position (4.93, 0.38, 20.2) μm with FWHM of 1.12 μm. Simultaneously, the 1.31 μm incident light dissipated, as shown in Figure 7c. When the GST in both sections changed to a crystalline state, the metalens completely turned off without any focusing, as shown in Figure 7d. The results show that metalens M_3_ can separate two different lights and focus them at different positions in horizontal direction. The output can be controlled on demand by turning the state of the GST in different sections.

**Figure 8:**
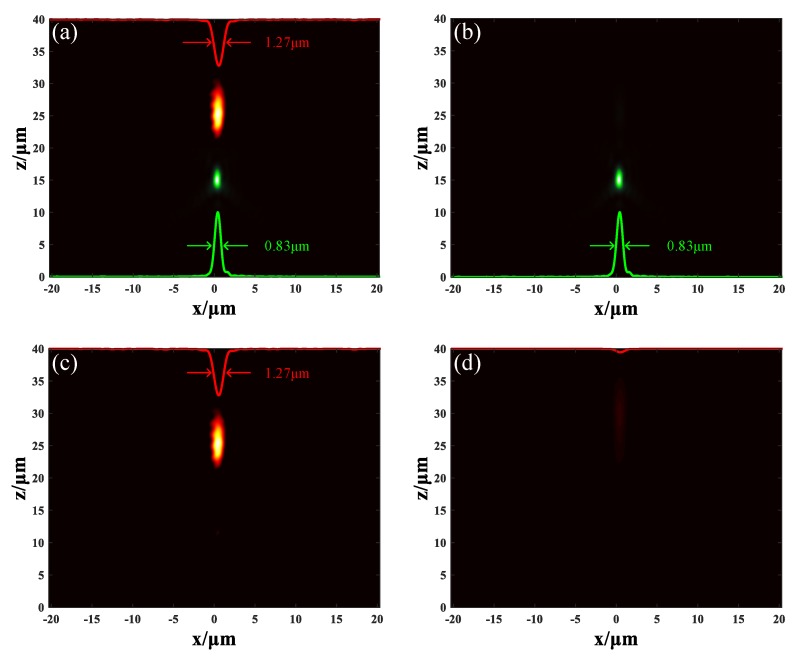
The light field distribution of metalens M_4_. (**a**) The GST in both sections are in amorphous state. (**b**) The GST in section A stays in amorphous state while the GST in section B stays in crystalline state. (**c**) The GST in section A stays in crystalline state while the GST in section B stays in amorphous state. (**d**) The GST in both sections are in crystalline state.

Considering metalens M_3_ separate the incident in horizontal, here we designed another metalens M_4_ to separate the incident light in vertical. M_4_ was also constructed by putting unit cells U_1_ and U_2_ together. Unit cells U_1_ were arranged in section A as *x_d_* = 0, *y_d_* = 0, *f* = 15 μm, and unit cells U_2_ were arranged in section B as *x_d_* = 0, *y_d_* = 0, *f* = 25 μm. The corresponding phase distribution of metalens M_4_ along the *x* axis is depicted in Figure 3f. The incident light remained the mixed light of two wavelengths at 1.55 μm and 1.31 μm. When the GST in both of the two sections was in an amorphous state, Figure 8a demonstrates how the incident light was focused at two different positions in a vertical direction: the 1.55 μm light was focused at the position (0.56, 0.56, 25.4) μm displayed as the red spot with FWHM of 1.27 μm; the 1.31 μm light was focused at the position (0.41, 0.41, 15) μm displayed as the green spot with FWHM of 0.83 μm. When the GST in section A remained in an amorphous state but the GST in section B is turned into a crystalline state, only the 1.31 μm light was focused at the position (0.41, 0.41, 15) μm with FWHM of 0.83 μm, as shown in Figure 8b. When the GST in section A was turned into crystalline state but the GST in section B stayed in an amorphous state, only the 1.55 μm light was focused at the position (0.56, 0.56, 25.4) μm with FWHM of 1.27 μm, as seen in Figure 8c. When the GST in both sections stayed in a crystalline state, Figure 8d shows that the metalens M_4_ was turned off. These results prove that the tunable duplex metalens M_4_ can divide the two different lights in a vertical direction and actively control them if necessary. The proposal of the metalens M_4_ may bring some reference value to three-dimensional colored imaging. Four metalenses (M_1_, M_2_, M_3_ and M_4_) have been designed to accomplish different functions in this article. The comparison of them can be seen in Table 1.

## 4. Conclusions

In this paper, by utilizing phase change material GST, we designed two types of unit cells to modulate the phase of incident light: when the GST stays in amorphous state, U_1_ responds to 1.31 μm light but shuts off at the wavelength of 1.55 μm. In contrast, U_2_ responds to 1.55 μm light but shuts off at the wavelength of 1.31 μm. When the GST stays in crystalline state, the two unit cells are shut off at either wavelength. On the basis of two other types of unit cells, four metalenses were designed with different functions. Metalens M_1_ and M_2_ can divide the single-wavelength light into two parts and focus them at different positions along horizontal and vertical directions, respectively. In comparison, the effects of metalenses can be actively controlled by tuning the state of GST on demand. Metalens M_3_ and M_4_ can separate the dual-wavelength light and focus them at designed positions along horizontal and vertical directions, respectively. By tuning the state of GST, the two metalenses can also achieve different focusing effects as necessary. In summary, our tunable duplex metalenses would make metalens more applicable in photonic devices.

## Figures and Tables

**Table 1:** Comparison of the four metalenses.

Metalens	M_1_	M_2_	M_3_	M_4_
Constructed unitcell	U_1_	U_1_	U_1_ and U_2_	U_1_ and U_2_
Function	Single-wavelength duplex	Single-wavelength duplex	Dual- wavelength duplex	Dual- wavelength duplex
Wavelength	1.31 μm	1.31 μm	1.31 μm and 1.55 μm	1.31 μm and 1.55 μm
Duplex direction	Horizontal	Vertical	Horizontal	Vertical
Tunability	Tunable	Tunable	Tunable	tunable
